# Persistent olfactory dysfunction associated with poor sleep quality and anxiety in patients with long COVID

**DOI:** 10.3389/fnins.2023.1161904

**Published:** 2023-05-12

**Authors:** Alna Carolina Mendes Paranhos, Apio Ricardo Nazareth Dias, Thalita da Rocha Bastos, Arthur Nascimento Rodrigues, Karem Harumy Yamamoto Santana, Lorena Henriete Araujo Dias, Lidiane Palheta Miranda dos Santos, Antônio José Cerasi, Michely Caroline Nascimento Mendes, Cleiziane Lima de Oliveira, Mariângela Moreno Domingues, Gisele Vieira Hennemann Koury, Pedro Fernando da Costa Vasconcelos, Givago Silva Souza, Juarez Antônio Simões Quaresma, Luiz Fábio Magno Falcão

**Affiliations:** ^1^Health and Biological Science Center, State University of Pará, Pará, Brazil; ^2^Tropical Medicine Center, Federal University of Pará, Pará, Brazil; ^3^Biological Science Center, Federal University of Pará, Pará, Brazil; ^4^Faculdade de Medicina, Universidade de São Paulo, São Paulo, Brazil

**Keywords:** long COVID, neurological manifestations, sleep disorders, olfaction disorders, anxiety

## Abstract

**Introduction:**

Poor sleep quality have been widely reported in patients with long COVID. Determining the characteristics, type, severity, and relationship of long COVID with other neurological symptoms is essential for the prognosis and management of poor sleep quality.

**Methods:**

This cross-sectional study was conducted at a public university in the eastern Amazon region of Brazil between November 2020 and October 2022. The study involved 288 patients with long COVID with self-report neurological symptoms. One hundred thirty-one patients were evaluated by using standardised protocols: Pittsburgh sleep quality index (PSQI), Beck Anxiety Inventory, Chemosensory Clinical Research Center (CCRC), and Montreal Cognitive Assessment (MoCA). This study aimed to describe the sociodemographic and clinical characteristics of patients with long COVID with poor sleep quality and their relationship with other neurological symptoms (anxiety, cognitive impairment, and olfactory disorder).

**Results:**

Patients with poor sleep quality were mainly women (76.3%), 44.04 ± 12.73 years old, with >12 years of education (93.1%), and had monthly incomes of up to US $240.00 (54.2%). Anxiety and olfactory disorder were more common in patients with poor sleep quality.

**Discussion:**

Multivariate analysis shows that the prevalence of poor sleep quality was higher in patients with anxiety, and olfactory disorder is associated with poor sleep quality. In this cohort of patients with long COVID, the prevalence of poor sleep quality was highest in the group tested by PSQI and were associated with other neurological symptoms, such as anxiety and olfactory dysfunction. A previous study indicates a significant association between poor sleep quality and psychological disorders over time. Recent studies involving neuroimaging found functional and structural changes in Long COVID patients with persistent olfactory disfunction. Poor sleep quality are integral part of complex changes related to Long COVID and should be part of patient’s clinical management.

## Introduction

Long COVID is a multisystem condition characterized by presence of signs and symptoms during or after COVID-19 that persisted for more than 4 weeks and which cannot be explained by an alternative diagnosis (Raveendran, 2021; Davis et al., 2023). Most patients diagnosed with long COVID were female (59.8%), was aged 36 to 50 (34.6%) and had not been hospitalized (75.8) (FAIR, 2022).

Sleep plays a vital role in maintaining mental and physical health; a single night of sleep deprivation can weaken the immune system and trigger other disorders (Ibarra-Coronado et al., 2015; Innocenti et al., 2020; El Sayed et al., 2021). Sleep quality is essential for memory consolidation, including sensory memory like taste and smell (Velluti, 1997; Barnes and Wilson, 2014). Determining the characteristics, type, severity, and relationships of long COVID with other symptoms is essential for the prognosis and management of poor sleep quality. This study describes the sociodemographic and clinical characteristics of patients with long COVID with persistent poor sleep quality following severe acute COVID-19 and their relationship with other symptoms (anxiety, cognitive impairment, and olfactory disorder).

## Materials and methods

### Ethical aspects

This study was conducted in accordance with ethical standards and the Helsinki declaration and its later amendments. The ethics and research committee of the State University of Pará (Belem, Brazil) approved this study (Opinion No. 4,252,664), and written informed consent was obtained from all participants included in the study.

### Study population and site

The study was conducted in accordance with the Strengthening the Reporting of Observational Studies in Epidemiology (STROBE) guidelines for reporting observational studies (Cuschieri, 2019) and was conducted on patients who were enrolled for a follow-up programme for long COVID at a public university in the eastern Amazon region, Brazil. The study participants included men and women ≥18 years old with long-term neurological complaints who underwent reverse transcriptase-polymerase chain reaction or serological testing.

**Figure 1:**
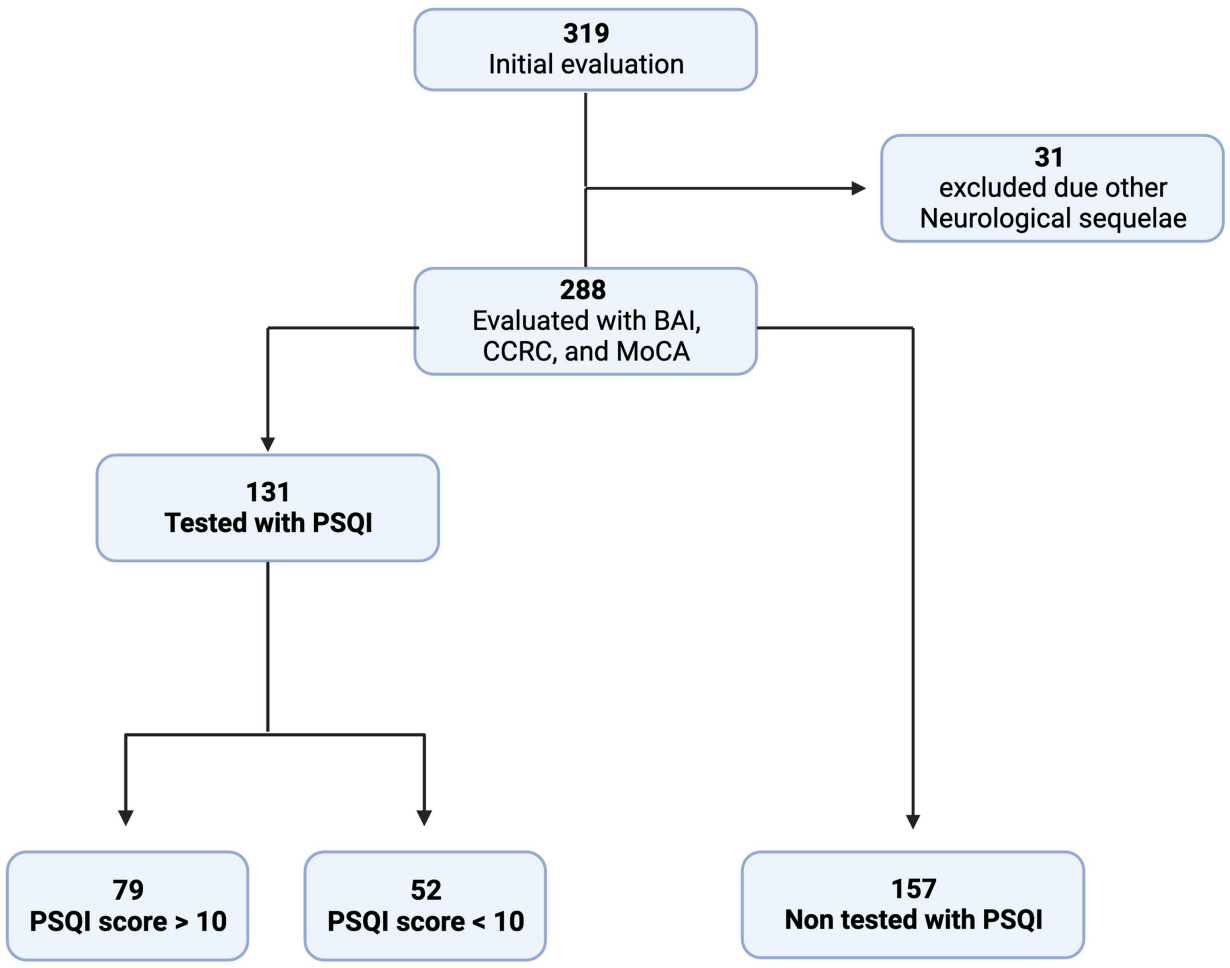
Flowchart of the study. BAI: Beck Anxiety Inventory. CCRC: Connecticut Chemosensory Research Center. MoCA: Montreal Cognitive Assessment. PSQI: Pittsburgh Sleep Quality Index.

Three hundred nineteen patients were contacted and evaluated medically (anamnesis and neurological tests) between November 2020 and October 2022. Of the 319 patients, 31 were excluded due to previous neurological sequelae. The remaining 288 patients were evaluated using the following diagnostic instruments: Beck Anxiety Inventory for diagnosis of anxiety disturbances, Chemosensory Clinical Research Center (CCRC) for olfactory evaluation, and Montreal Cognitive Assessment (MoCA) for cognitive evaluation. Furthermore, 131 patients with complaints of sleep quality following severe acute COVID-19 were evaluated using the Pittsburgh sleep quality index (PSQI) for sleep quality evaluation. The PSQI evaluation results and this group’s clinical data were compared to 157 patients without sleep complaints (Figure 1).

### Study design, data collection, and procedures

This is an observational, cross-sectional study. A standardised evaluation form was used to collect sociodemographic and clinical data. The form contains data on education, sex, monthly income, and all symptoms associated with long-term COVID (such as headache, ageusia, fatigue, dyspnoea, myalgia, chest pain, back pain) and poor sleep quality that started after COVID-19 infection that could not be explained by other factors. Patients with suspected long-term COVID-related fatigue were evaluated by a multidisciplinary team and a neurologist regarding the nature of the symptoms, the time of onset and their impact on functional status. Aspects of premorbid and intercurrent mental health, mainly in relation to symptoms of depression, anxiety and post-traumatic stress disorder were also collected during the clinical interview. A short physical examination was conducted to assess pulmonary and cardiac functions and neurological findings.

The monthly income assessment was included in our initial interview as part of the socio-demographic data, the objective was to collect data that help answer possible iterations between the socioeconomic level and the development of long COVID. Such data is especially important in treatment of patients from the public health system in Brazil. The period for the calculation was the month referring to the date of the interview, first categorized into ranges based on the current minimum wage in Brazil and subsequently converted into US dollars.

The PSQI was used to evaluate sleep quality. The use of the PSQI followed the original recommendations (Buysse et al., 1998) in the Brazilian version (Bertolazi et al., 2011) with regard to sleep quality from the preceding month. Each patient answered 19 questions separated into seven components that included sleep quality, sleep latency, sleep duration, habitual sleep efficiency, poor sleep quality, sleeping medication, and daytime dysfunction. Each component had scores ranging from 0–3, with a total score of 21 points. Sleep quality staging followed the following classification: A PSQI score < 5 indicate a good sleep quality, and a PSQI score > 5 indicate a poor sleep quality. Patients with a PSQI global score > 5 indicates that the individual is having severe difficulties in at least two areas, or moderate difficulties in more than three areas. The global score is therefore “transparent,” i.e., it conveys information about the severity of the individual’s problem, and the number of problems present, through a single simple measure (Buysse et al., 1998).

Patients were grouped in two groups: individuals non-tested by PSQI and individuals tested by PSQI that clinical characteristics were compared. The individuals tested by PSQI was subdivided in PSQI score ≤ 10, and PSQI > 10, that were compared. Univariate and multivariate logistic regression analyses were performed to define the associated odds ratio between the poor sleep quality, epidemiological and clinical characteristics of the sample: sex, hospitalization, duration of long COVID symptoms, anxiety, mild cognitive impairment, olfactory disorder, ageusia, fatigue, and dyspnoea.

### Statistical analysis

The collected data were tabulated in a Microsoft Excel^™^ spreadsheet (Microsoft Inc., Richmond, WA, United States). The GraphPad Prism software version 6.0.1^™^ (GraphPad, San Diego, CA, United States) was used for statistical analysis. D’Agostino–Pearson test was used to determine the normality of samples. Continuous variables data were presented as mean ± standard deviation and categorical variables were presented as absolute and relative frequencies. Student *t*-test was used to access parametric data. Non-parametric data were assessed using the Wilcoxon test. The categorical variables were assessed by the chi-square test or Fisher’s exact test, as appropriate. A Binary logistic regression analysis was performed. The association between the patient’s exposure factors (female sex, hospitalization, time from symptoms onset, fatigue, dyspnoea, anxiety, olfactory disorder, ageusia, mild cognitive impairment) and the outcome (poor sleep quality) was tested, with the calculation of raw odds ratios (OR) for each exposure factor, and respective confidence intervals (CIs). The arrangement with a better calculated *r*^2^ was considered. An alpha level of 5% (*p* < 0.05) was adopted to reject the null hypothesis.

## Results

The patients tested by PSQI were mainly women *n* = 100 (76.3%), 44.04 ± 12.73 years old (mean ± standard deviation), with >12 years of education *n* = 122 (93.1%), and had monthly incomes of up to US $240.00 (54.2%). Only 17 (12.9%) patients were admitted to the hospital during their acute phases of COVID. Their mean duration of symptoms was 265.66 ± 144.42 days, which was not statistically different from the group with no sleep complaints. Anxiety [non-tested by PSQI group *n* (%)/tested by PSQI group *n* (%), *p*-value 74 (47)/105 (80), 0.000], and olfactory disorder [non-tested by PSQI group *n* (%)/tested by PSQI group *n* (%), *p*-value 66 (42)/82 (62), *p*-value 0.0005] were symptoms that were more frequently found among patients in the group tested by PSQI (Table 1).

**Table 1:** Sociodemographic and clinical characteristics of patients evaluated by groups (*n* = 288).

Variable	General (*n* = 288)	Non-tested by PSQI (*n* = 157)	Tested by PSQI (*n* = 131)	*p*-value
**Sex**				
Female, *n* (%)	218 (75.7)	118 (75.2)	100 (76.3)	0.81
Male, *n* (%)	70 (24.3)	39 (24.8)	31 (23.7)	
**Age**	45.53 ± 13.15	46.78 ± 13.40	44.04 ± 12.73	0.07
**Years of study**				
Up to 9 years, *n* (%)	26 (9)	17 (10.8)	9 (6.8)	0.24
12 years or more, *n* (%)	262 (91)	140 (89.2)	122 (93.2)	
**Monthly income**				
Up to US$ 240.00, *n* (%)	156 (54.2)	85 (54.1)	71 (54.2)	0.99
More than US$ 240.00	132 (45.8)	72 (45.9)	60 (45.8)	
**Clinical data**				
Hospital admittance, *n* (%)	43 (14.9)	26 (16.5)	17 (12.9)	0.39
Time from onset symptoms	300.28 ± 201.30	329.16 ± 235.21	265.66 ± 144.42	0.45
**Self-related symptoms**				
Headache, *n* (%)	137 (47.6)	79 (50.3)	58 (44.3)	0.30
Ageusia, *n* (%)	129 (44.8)	66 (42)	63 (48)	0.30
Fatigue, *n* (%)	137 (47.5)	77 (49)	60 (45.8)	0.58
Dyspnoea, *n* (%)	65 (22.6)	39 (24.8)	26 (19.8)	0.31
Myalgia, *n* (%)	81 (28.1)	47 (29.9)	34 (25.9)	0.45
Chest pain, *n* (%)	49 (17)	30 (19.1)	19 (14.5)	0.30
Back pain, *n* (%)	63 (21.8)	35 (22.3)	28 (21.4)	0.85
**Measured symptoms**				
Anxiety (BAI) *n* (%)	179 (62.1)	74 (47)	105 (80)	0.000^#^
Olfactory disorder (CCRC), *n* (%)	148 (51.3)	66 (42)	82 (62)	0.0005^#^
Mild cognitive disorder (MoCA), *n* (%)	129 (44.7)	65 (41)	64 (48)	0.20

PSQI, Pittsburgh sleep quality index; BAI, Beck Anxiety Inventory; CCRC: Connecticut Chemosensory Research Center; MoCA: Montreal Cognitive Assessment. ^#^Chi-square (*p*-value < 0.05).

The evaluation of sleep quality using PSQI was undertaken in patients with and without self-related sleep complaints, and the results show that *n* = 114 (89.06%) was bad sleepers (PSQI score > 5) and from these, *n* = 77 (60,15%) had a PSQI score > 10, that indicates severe difficulties for sleep. In patients with severe difficulties for sleep, the period until sleep onset was >60 min *n* = 47 (59.5), and their sleep durations were short (mean ± standard deviation no poor sleep quality/poor sleep quality 6.34 ± 1.31/4.92 ± 1.22, *p*-value 0.000). The administration of sleeping pills was reported by *n* = 38 (48%) of the patients with severe difficulties for sleep, and this group reported more problems keeping up with enthusiasm for daily activities [*n* (%) no poor sleep quality/poor sleep quality 35 (67.3)/75(95), *p*-value 0.000]. The self-reported reasons for trouble sleeping were due to getting up to use the bathroom *n* = 69 (87.3), having pain *n* = 60 (75.9), feeling too hot *n* = 58 (73.4), having bad dreams *n* = 52 (65.8), and not being able to breathe comfortably *n* = 49 (62) (Table 2).

**Table 2:** PSQI components of patients with sleep complaints by group (*n* = 131).

	General (*n* = 131)	PSQI score ≤ 10 (*n* = 52)	PSQI score > 10 (*n* = 79)	*p*-value
**Time to fall asleep, *n* (%)**				
<15 min, *n* (%)	15 (11.5)	11 (21.2)	4 (5)	**0.004**^**#**^
16–30 min, *n* (%)	31 (23.6)	24 (46)	7 (8.9)	**0.000**^**#**^
31–60 min, *n* (%)	33 (25.2)	12 (23)	21 (26.6)	0.65
>60 min, *n* (%)	52 (39.7)	5 (9.8)	47 (59.5)	**0.000**^**#**^
**Hours of actual sleep (mean ± SD)**	5.49 ± 1.43	6.34 ± 1.31	4.92 ± 1.22	0.000*
**Use of sleeping pills, *n* (%)**	42 (32)	4 (7.7)	38 (48)	**0.000**^**#**^
**Difficulties due to insomnia, *n* (%)**				
Get to sleep within 30 min	114 (87)	40 (76.9)	74 (93.7)	**0.005**^**#**^
Staying awake during activities	99 (75.6)	38 (73)	61 (77.2)	0.58
Keep up enthusiasm	110 (83.9)	35 (67.3)	75 (95)	**0.000**^**#**^
**Causes of insomnia, *n* (%)**				
Wake up in the middle of the night	118 (90)	44 (84.6)	74 (93.7)	0.08
Get up to use the bathroom	107 (81.7)	38 (73)	69 (87.3)	**0.03**^**#**^
Have pain	88 (67.2)	28 (53.8)	60 (75.9)	**0.008**^**#**^
Feel too hot	71 (54.2)	13 (25)	58 (73.4)	**0.000**^**#**^
Have bad dreams	69 (52.7)	17 (32.7)	52 (65.8)	**0.000**^**#**^
Cannot breathe comfortably	70 (53.4)	21 (40.4)	49 (62)	**0.015**^**#**^
Cough or snore	63 (48)	20 (38.5)	43 (54.4)	0.07
Feel too cold	62 (47.3)	20 (38.5)	42 (53)	0.09
Others	76 (58)	24 (46)	52 (65.8)	**0.02**^**#**^

The bold values indicate the *p*-values with values minor or equal 0.05. PSQI: Pittsburgh sleep quality index. *Mann–Whitnney (*p*-value < 0.05), ^#^Qui-quadrado (*p*-value < 0.05).

Logistic regression analysis showed there was a significant odds ratio of poor sleep quality among women [OR (CI-95%) 1.93 (1–3.70), *p*-value 0.04] in univariate analysis, and [OR (CI-95%) 2.14 (1.01–4.51), *p*-value 0.04] in multivariate analysis. The prevalence of poor sleep quality was higher in patients with anxiety [OR (CI-95%) 8.19 (3.89–17.24), *p*-value 0.000] in univariate analysis, and [OR (CI-95%) 8.62 (3.89–19.12), *p*-value 0.000] in multivariate analysis. The olfactory disorder is associated with poor sleep quality [OR (CI-95%) 2.20 (1.19–4.07), *p*-value 0.01] in multivariate analysis (Table 3).

**Table 3:** The association between poor sleep quality and clinical features of the study population (*n* = 288).

Clinical feature	Univariate analysis		Multivariate analysis	
	OR (95% CI)	*p*-value	OR (95% CI)	*p*-value
Female	1.93 (1–3.70)	**0.04**	2.14 (1.01–4.51)	**0.04**
Hospitalization	0.91 (0.44–1.87)	0.80	0.82 (0.35–1.92)	0.64
Symptom onset (>6 months)	0.78 (0.44–1.39)	0.41	0.71 (0.37–1.36)	0.30
Fatigue	1.13 (0.63–2.00)	0.67	1.21 (0.58–2.50)	0.60
Dyspnoea	0.91 (0.49–1.70)	0.78	0.95 (0.44–2.02)	0.89
Anxiety (BAI)	8.19 (3.89–17.24)	**0.000**	8.62 (3.89–19.12)	**0.000**
Olfactory disorder (CCRC)	1.53 (0.91–2.55)	0.10	2.20 (1.19–4.07)	**0.01**
Ageusia	0.85 (0.51–1.43)	0.55	0.66 (0.36–1.29)	0.24
Mild cognitive impairment (MoCA)	1.09 (0.62–1.91)	0.75	1.39 (0.70–2.74)	0.34

The bold values indicate the *p*-values with values minor or equal 0.05. BAI, Anxiety Index; CCRC, Connecticut Chemosensory Research Center; MoCA, Montreal Cognitive Assessment.

## Discussion

In this cross-sectional study of 288 patients with long COVID and self-reported neurological symptoms, 131 (45.5%) patients had sleep complaints. Of these, 79 (27%) were diagnosed with poor sleep quality, according to PSQI. The group with poor sleep quality was mainly composed of women (between 44.04 ± 12.73 years), with ≥12 years of education and no related hospital admissions. Our analysis of the PSQI components showed that the group with poor sleep quality slept for fewer hours per night (4.92 ± 1.22/6.34 ± 1.31, *p* = 0.000) compared to no poor sleep quality group, had a high frequency of sleep pills utilization [38 (48%)/4 (7.7), *p* = 0.000] and less enthusiasm to get things done [75 (95%)/35 (67.3), *p* = 0.000] than individuals with no poor sleep quality. The group with poor sleep quality had more anxiety and olfactory dysfunction symptoms than the group without sleep orders. In a regression analysis, anxiety [8.62 (3.89–19.12), *p* = 0.000], olfactory dysfunction [2.20 (1.19–4.07), *p* = 0.01] and female sex [2.14 (1.01–4.51), *p* = 0.04] were risks factors associated with poor sleep quality in this population.

The 27% prevalence of poor sleep quality in our sample group was consistent with those found in two reviews and meta-analysis studies involving post-COVID sequelae (27%) (Groff et al., 2021) and (32.9%) (Wu et al., 2021). Previous studies established that poor sleep quality is one of the most prevalent neurological symptoms among COVID-19 survivors, affecting approximately one-third of the population (Moura et al., 2022; Pinzon et al., 2022), particularly women, young people, and patients with mood disorders (Ahmed et al., 2021; Mendes Paranhos et al., 2022). A recent study showed that 73.8% of patients with long COVID and poor sleep quality were women, and this sex difference in poor sleep quality may be associated with hormonal factors (Goweda et al., 2020). Furthermore, women tend to seek health services more regularly, possibly contributing to greater diagnosis in this group (Wang et al., 2013; Fernández-de-Las-Peñas et al., 2022).

In our analysis, the most affected PSQI components in the group diagnosed with poor sleep quality were difficulty initiating sleep, less sleep duration, administration of sleeping pills, nycturia, pain, nightmares, nocturnal breathing problems, feeling too hot, and less enthusiasm to get things done. It is well known that almost all of these components are associated with anxiety symptoms, which affect more than half of the population (Cutler, 2016; Oh et al., 2019). Long-term COVID-related poor sleep quality were associated with neuroinflammation and psychological disorders in a follow-up study with previously hospitalized patients with COVID-19 (Pellitteri et al., 2022). The patients were evaluated at 2 months (T1) and 10 months (T2) after discharge. The results showed the increased prevalence of insomnia of 10.6% in baseline to 27.3% at 10 months (T2), and a significant association between T2 PSQI total score and T2 anxiety levels, suggesting an association between poor sleep quality and psychological disorders over time.

Similar results were found in our univariate and multivariate logistic regression analyses, where poor sleep quality and anxiety in the sample population were associated with a higher odds ratio and could be correlated. These two symptoms were associated with high comorbidity in patients with long COVID. A comorbid mental health condition, such as anxiety and depressive disorders, affects 40% of patients with insomnia, and the onset of these conditions can be predicted by features listed in the diagnostic and statistical manual of mental disorders (DSM-5) (Roth, 2007; Huang and Zhao, 2020; Bard et al., 2023). Additionally, insomnia and anxiety have a hyperarousal pathogenetic mechanism caused by the dysregulation of neurotransmitter systems, including cholinergic and gamma-aminobutyric acid (GABA) (Blake et al., 2018). Hyperarousal and insufficient sleep disrupt the corticolimbic circuitry function, impairing affective reactivity and regulation (Riemann et al., 2010).

The higher odds ratio in our study, which could indicate the correlation between the two objectively measured outcomes of olfactory dysfunction and the occurrence of poor sleep quality in the patients evaluated, was a significant finding. These sleep and olfactory disturbances are commonly associated with various pathologies, including Alzheimer’s disease, Parkinson’s disease, schizophrenia, and depression (Barresi et al., 2012). In context of COVID-19 patients, recent studies involved neuroimaging found functional and structural changes in long COVID patients with persistent olfactory dysfunction such as presence of microhemorrhages at olfactory bulb (Aragão et al., 2020) and olfactory bulb edema (Laurendon et al., 2020); reduced tissue perfusion in the orbital and medial frontal regions (Yus et al., 2022); decreased in grey matter (GM) volume and increased in mean diffusivity in olfactory related regions (Wingrove et al., 2023); increased in functional connectivity (FC) between the left orbitofrontal cortex (OFC), visual association cortex and cerebellum and reductions between the right (OFC) and dorsal anterior cingulate cortex (Campabadal et al., 2023). These data support the hypothesis that persistent olfactory dysfunction may reduce attentional processing towards olfactory stimuli and perhaps the sustained lack of olfactory attention or sensing underlies, which might explain why some COVID-19 patients have not recovered their sense of smell, and olfactory impairment as a potential biomarker of subsequent neurodegeneration (Campabadal et al., 2023).

A wide variety of information captured in the waking period depends on sleep to be consolidated, including sensory memory (Barnes and Wilson, 2014). Previous findings regarding smell have shown that sleep favours changes in olfactory cortical circuits contributing to the strength and precision of odour memories and perception (Miyamoto et al., 2009; Barnes and Wilson, 2014). During sleep, especially during slow-wave sleep (SWP), the piriform cortex becomes hypo-responsive to environmental odour stimulation. It enhances functional connectivity between other cortical regions and the limbic system, compared to the waking state (Günbey et al., 2015). For example, a common behavioural response in many mammals is post-prandial sleep, which contributes to the memory of odours and flavours of consumed food (Yokoyama et al., 2011). Poor sleep quality are integral part of complex changes related to long COVID and should be part of the patient’s clinical management.

In our findings cognitive impairment in long COVID patients (assessed by MoCA) was not associated with sleep problems. A previous study with a more comprehensive neuropsychological protocol showed that cognitive performance was correlated with olfactory dysfunction, PSQI had moderate correlations with processing speed and letter fluency, anxiety to a lesser extent, but not depression. The authors argue that cognitive disorder is not secondary to psychological aspects, consistent with our results (Delgado-Alonso et al., 2022).

This study has some limitations. First, the single-center cross-sectional design of the study limits the generalisability of the data, and all inferences about causality and effect are hypothetical. Moreover, the absence of formal data regarding previous clinical history and the acute phase of COVID-19 is a potential confounding factor, which was minimized by carefully using an anamnesis form and specialized consultation with neurologists. The self-related symptoms, including sleep orders and other limitations. These were assessed in the search form and were part of a qualitative sample characterisation. The use of quantitative tools is necessary for more precision. Future follow-up and intervention studies should be conducted to monitor this population and assess the effectiveness of treatments. From a clinical point of view, we recommend screening patients with acute and post-acute COVID-19 for poor sleep quality associated with mood disorders and olfactory dysfunction to improve appropriate treatment. The use of polysomnography to monitor and assess any potential obstructive respiratory events that might affect sleep quality is recommended.

## Conclusion

A high prevalence of individuals with long-term poor sleep quality was observed in this cohort of patients with long COVID and associated neurological symptoms, such as anxiety and olfactory dysfunction. Our results highlight the need to continue monitoring the rate of associated neurological symptoms in long COVID over time. Furthermore, clinical trials and longitudinal studies are recommended to verify the effectiveness of potential treatments and the postulated risk for an increase in neurodegenerative disorders in this population.

## Data availability statement

The original contributions presented in the study are included in the article/supplementary material, further inquiries can be directed to the corresponding author.

## Ethics statement

The studies involving human participants were reviewed and approved by Comitê de Ética em Pesquisa com Seres Humanos do Centro de Ciências Biológicas e da Saúde, Universidade do Estado do Pará. The patients/participants provided their written informed consent to participate in this study.

## Author contributions

AP, AD, GS, JQ, and LF: design and conduct of the study. AP, AC, TB, AR, KS, LD, LS, MM, CO, MD, and GK: collection, management, analysis, and interpretation of the data. GS, JQ, LF, PV, AP, and AD: preparation, review, or approval of the manuscript. All authors contributed to the article and approved the submitted version.

## Funding

This study was funded by Fundação Amazônia de Amparo a Estudos e Pesquisa (FAPESPA 006/2020), Secretaria de Estado de Ciência, Tecnologia e Educação Técnica e Tecnológica (SECTET 09/2021), Coordination for the improvement of Higher Education Personnel (CAPES PDPG AMAZÔNIA LEGAL), and National Council for Scientific and Technological Development (CNPq).

## Conflict of interest

The authors declare that the research was conducted without any commercial or financial relationships that could be construed as a potential conflict of interest.

## Publisher’s note

All claims expressed in this article are solely those of the authors and do not necessarily represent those of their affiliated organizations, or those of the publisher, the editors and the reviewers. Any product that may be evaluated in this article, or claim that may be made by its manufacturer, is not guaranteed or endorsed by the publisher.

## References

[ref1] Ahmed G. K. Khedr E. M. Hamad D. A. Meshref T. S. Hashem M. M. Aly M. M. (2021). Long term impact of COVID-19 infection on sleep and mental health: a cross-sectional study. Psychiatry Res. 305:114243. doi: 10.1016/j.psychres.2021.114243, PMID: 34673325 PMC8507572

[ref2] Akinci T. Basar H. M. (2021). Relationship between sleep quality and the psychological status of patients hospitalized with COVID-19. Sleep Med. 80, 167–170. doi: 10.1016/j.sleep.2021.01.034, PMID: 33601228 PMC7842153

[ref3] Alrasheed M. M. Afnan M. A. Khulood B. S. Sinaa A. Haitham A. J. Ahmed S. B. (2021). The impact of quarantine on sleep quality and psychological distress during the COVID-19 pandemic. Nat. Sci. Sleep 13, 1037–1048. doi: 10.2147/NSS.S313373, PMID: 34262375 PMC8273741

[ref4] Altena E. Baglioni C. Espie C. A. Ellis J. Gavriloff D. Holzinger B.. (2020). Dealing with sleep problems during home confinement due to the COVID-19 outbreak: practical recommendations from a task force of the European CBT-I Academy. J. Sleep Res. 29, 1–7. doi: 10.1111/jsr.13052 32246787

[ref5] Aragão M. F. V. V. Leal M. C. Cartaxo Filho O. Q. Fonseca T. M. Valença M. M. (2020). Anosmia in COVID-19 associated with injury to the olfactory bulbs evident on MRI. Am. J. Neuroradiol. 41, 1703–1706. doi: 10.3174/ajnr.A6675 32586960 PMC7583088

[ref6] Bacaro V. Chiabudini M. Buonanno C. De Bartolo P. Riemann D. Mancini F.. (2020). Insomnia in the Italian population during COVID-19 outbreak: a snapshot on one major risk factor for depression and anxiety. Front Psychiatry 11:579107. doi: 10.3389/fpsyt.2020.579107, PMID: 33384625 PMC7769843

[ref7] Bard H. A. O’Driscoll C. Miller C. B. Henry A. L. Cape J. Espie C. A. (2023). Insomnia, depression, and anxiety symptoms interact and individually impact functioning: a network and relative importance analysis in the context of insomnia. Sleep Med. 101, 505–514. doi: 10.1016/j.sleep.2022.12.005, PMID: 36527942

[ref8] Barnes D. C. Wilson D. A. (2014). Sleep and olfactory cortical plasticity. Front. Behav. Neurosci. 8:134. doi: 10.3389/fnbeh.2014.00134, PMID: 24795585 PMC4001050

[ref9] Barresi M. Ciurleo R. Giacoppo S. Foti Cuzzola V. Celi D. Bramanti P.. (2012). Evaluation of olfactory dysfunction in neurodegenerative diseases. J. Neurol. Sci. 323, 16–24. doi: 10.1016/j.jns.2012.08.028 23010543

[ref10] Beck F. Leger D. Cortaredona S. Verger P. Peretti-Watel P. (2021). Would we recover better sleep at the end of COVID-19? A relative improvement observed at the population level with the end of the lockdown in France. Sleep Med. 78, 115–119. doi: 10.1016/j.sleep.2020.11.029, PMID: 33422813 PMC7722490

[ref11] Bertolazi A. N. Fagondes S. C. Hoff L. S. Dartora E. G. da Silva Miozzo I. C. de Barba M. E. F.. (2011). Validation of the Brazilian Portuguese version of the Pittsburgh sleep quality index. Sleep Med. 12, 70–75. doi: 10.1016/j.sleep.2010.04.020, PMID: 21145786

[ref12] Blake M. J. Trinder J. A. Allen N. B. (2018). Mechanisms underlying the association between insomnia, anxiety, and depression in adolescence: implications for behavioral sleep interventions. Clin. Psychol. Rev. 63, 25–40. doi: 10.1016/j.cpr.2018.05.006, PMID: 29879564

[ref13] Blume C. Schmidt M. H. Cajochen C. (2020). Effects of the COVID-19 lockdown on human sleep and rest-activity rhythms. Curr. Biol. 30, R795–R797. doi: 10.1016/j.cub.2020.06.021 32693067 PMC7284244

[ref14] Buysse D. J. Reynolds C. F. Monk T. H. Berman S. R. Kupfer D. J. (1998). The Pittsburgh sleep quality index: a new instrument for psychiatric practice and research. Psychiatry Res. 28, 193–213. doi: 10.1016/0165-1781(89)90047-4 2748771

[ref15] Campabadal A. Oltra J. Junqué C. Guillen N. Botí M. Á. Sala-Llonch R.. (2023). Structural brain changes in post-acute COVID-19 patients with persistent olfactory dysfunction. Ann. Clin. Transl. Neurol. 10, 195–203. doi: 10.1002/acn3.51710, PMID: 36525472 PMC9878006

[ref16] Cénat J. M. Blais-Rochette C. Kokou-Kpolou C. K. Noorishad P. G. Mukunzi J. N. McIntee S. E.. (2021). Prevalence of symptoms of depression, anxiety, insomnia, posttraumatic stress disorder, and psychological distress among populations affected by the COVID-19 pandemic: a systematics review and meta-analysis. Psychiatry Res. 295:113599. doi: 10.1016/j.psychres.2020.113599, PMID: 33285346 PMC7689353

[ref17] Cuschieri S. (2019). The STROBE guidelines. Saudi J. Anaesth. 13, S31–S34. doi: 10.4103/sja.SJA_543_18, PMID: 30930717 PMC6398292

[ref18] Cutler A. J. (2016). The role of insomnia in depression and anxiety. J. Clin. Psychiatry 77:e1010. doi: 10.4088/JCP.14076tx3c 27561147

[ref19] Davis H. E. Assaf G. S. McCorkell L. We H. Low R. J. Re’em Y.. (2021). Characterizing long COVID in an international cohort: 7 months of symptoms and their impact. EClinicalMedicine 38:101019. doi: 10.1016/j.eclinm.2021.101019, PMID: 34308300 PMC8280690

[ref20] Davis H. E. McCorkell L. Vogel J. M. Topol E. J. (2023). Long COVID: major findings, mechanisms and recommendations. Nat. Rev. Microbiol. 21, 133–146. doi: 10.1038/s41579-022-00846-2, PMID: 36639608 PMC9839201

[ref21] Delgado-Alonso C. Valles-Salgado M. Delgado-Álvarez A. Yus M. Gómez-Ruiz N. Jorquera M.. (2022). Cognitive dysfunction associated with COVID-19: a comprehensive neuropsychological study. J. Psychiatr. Res. 150, 40–46. doi: 10.1016/j.jpsychires.2022.03.033, PMID: 35349797 PMC8943429

[ref22] El Sayed S. Gomaa S. Shokry D. Kabil A. Eissa A. (2021). Sleep in post-COVID-19 recovery period and its impact on different domains of quality of life. Egypt J. Neurol. Psychiatry Neurosurg. 57:172. doi: 10.1186/s41983-021-00429-7, PMID: 34924750 PMC8669420

[ref23] FAIR Health (2022). Patients Diagnosed with Post-COVID Conditions: An Analysis of Private Healthcare Claims Using the Official ICD-10 Diagnostic Code. New York, NY: FAIR Health, Inc.

[ref24] Felician J. Galvao F. Lefebvre M. Nourredine M. Peter-Derex L. (2022). Association of delayed sleep/wake rhythm with depression during the first COVID-19 lockdown in France. Nat. Sci. Sleep 14, 1545–1557. doi: 10.2147/NSS.S369859, PMID: 36081862 PMC9447448

[ref25] Fernández-de-Las-Peñas C. Martín-Guerrero J. D. Pellicer-Valero Ó. J. Navarro-Pardo E. Gómez-Mayordomo V. Cuadrado M. L.. (2022). Female sex is a risk factor associated with long-term post-COVID related-symptoms but not with COVID-19 symptoms: the LONG-COVID-EXP-CM multicenter study. J. Clin. Med. 11:413. doi: 10.3390/jcm11020413, PMID: 35054108 PMC8778106

[ref26] Goweda R. A. Hassan-Hussein A. Alqahtani M. A. Janaini M. M. Alzahrani A. H. Sindy B. M.. (2020). Prevalence of poor sleep quality among medical students of Umm Al-Qura University, Makkah, Kingdom of Saudi Arabia. J. Public Health Res. 9:2020. doi: 10.4081/jphr.2020.1921 33575230 PMC7868773

[ref27] Groff D. Sun A. Ssentongo A. E. Ba D. M. Parsons N. Poudel G. R.. (2021). Short-term and long-term rates of postacute sequelae of SARS-CoV-2 infection: a systematic review. JAMA Netw. Open 4:e2128568. doi: 10.1001/jamanetworkopen.2021.28568, PMID: 34643720 PMC8515212

[ref28] Günbey E. Güzel A. Karlı R. Ünal R. (2015). The relationships between the clinical and polysomnographic findings and the olfactory function in patients with obstructive sleep apnea syndrome. Sleep Breath. 19, 1301–1307. doi: 10.1007/s11325-015-1165-3, PMID: 25855470

[ref29] Huang Y. Zhao N. (2020). Generalized anxiety disorder, depressive symptoms and sleep quality during COVID-19 outbreak in China: a web-based cross-sectional survey. Psychiatry Res. 288, 112954–112956. doi: 10.1016/j.psychres.2020.112954 32325383 PMC7152913

[ref30] Ibarra-Coronado E. G. Pantaleón-Martínez A. M. Velazquéz-Moctezuma J. Prospéro-García O. Méndez-Díaz M. Pérez-Tapia M.. (2015). The bidirectional relationship between sleep and immunity against infections. J Immunol Res 2015, 1–14. doi: 10.1155/2015/678164, PMID: 26417606 PMC4568388

[ref31] Innocenti P. Puzella A. Mogavero M. P. Bruni O. Ferri R. (2020). Letter to editor: COVID-19 pandemic and poor sleep quality-a web survey in Italy. Neurol. Sci. 41, 2021–2022. doi: 10.1007/s10072-020-04523-1, PMID: 32607852 PMC7324906

[ref32] Jinglong Z. Rong S. Juan Y. (2020). Anxiety and depression in elderly patients during epidemic of coronavirus disease 2019 and its influencing factors. J. Clin. Med. Pract. 04, 246–250.

[ref33] Laurendon T. Radulesco T. Mugnier J. Gérault M. Chagnaud C. El Ahmadi A. A.. (2020). Bilateral transient olfactory bulb edema during COVID-19-related anosmia. Neurology 95, 224–225. doi: 10.1212/WNL.0000000000009850, PMID: 32444492

[ref34] Lopez-Leon S. Wegman-Ostrosky T. Perelman C. Sepulveda R. Rebolledo P. A. Cuapio A.. (2021). More than 50 long-term effects of COVID-19: a systematic review and meta-analysis. Sci. Rep. 11:16144. doi: 10.1038/s41598-021-95565-8, PMID: 34373540 PMC8352980

[ref35] Mendes Paranhos A. C. Nazareth Dias Á. R. Machado da Silva L. C. Vieira Hennemann Koury G. de Jesus Sousa E. Cerasi A. J. Jr.. (2022). Sociodemographic characteristics and comorbidities of patients with long COVID and persistent olfactory dysfunction. JAMA Netw. Open 5:e2230637. doi: 10.1001/jamanetworkopen.2022.30637, PMID: 36074464 PMC9459661

[ref36] Miyamoto T. Miyamoto M. Iwanami M. Suzuki K. Inoue Y. Hirata K. (2009). Odor identification test as an indicator of idiopathic REM sleep behavior disorder. Mov. Disord. 24, 268–273. doi: 10.1002/mds.22361 18972547

[ref37] Moura A. E. F. Oliveira D. N. Torres D. M. Tavares-Júnior J. W. L. Nóbrega P. R. Braga-Neto P.. (2022). Central hypersomnia and chronic insomnia: expanding the spectrum of poor sleep quality in long COVID syndrome—a prospective cohort study. BMC Neurol. 22:417. doi: 10.1186/s12883-022-02940-7, PMID: 36352367 PMC9643986

[ref38] Oh C. M. Kim H. Y. Na H. K. Cho K. H. Chu M. K. (2019). The effect of anxiety and depression on sleep quality of individuals with high risk for insomnia: a population-based study. Front. Neurol. 10:849. doi: 10.3389/fneur.2019.00849, PMID: 31456736 PMC6700255

[ref39] Pellitteri G. Surcinelli A. De Martino M. Fabris M. Janes F. Bax F.. (2022). Sleep alterations following COVID-19 are associated with both neuroinflammation and psychological disorders, although at different times. Front. Neurol. 13:929480. doi: 10.3389/fneur.2022.929480, PMID: 36062000 PMC9428349

[ref40] Pinzon R. T. Wijaya V. O. Jody A. A. Nunsio P. N. Buana R. B. (2022). Persistent neurological manifestations in long COVID-19 syndrome: a systematic review and meta-analysis. J. Infect. Public Health 15, 856–869. doi: 10.1016/j.jiph.2022.06.013, PMID: 35785594 PMC9221935

[ref41] Raveendran A. V. (2021). Long COVID-19: challenges in the diagnosis and proposed diagnostic criteria. Diabetes Metab. Syndr. 15, 145–146. doi: 10.1016/j.dsx.2020.12.025, PMID: 33341598 PMC7737559

[ref42] Riemann D. Spiegelhalder K. Feige B. Voderholzer U. Berger M. Perlis M.. (2010). The hyperarousal model of insomnia: a review of the concept and its evidence. Sleep Med. Rev. 14, 19–31. doi: 10.1016/j.smrv.2009.04.002, PMID: 19481481

[ref43] Roth T. (2007). Insomnia: definition, prevalence, etiology, and consequences. J. Clin. Sleep Med. 3, S7–S10. PMID: 17824495 PMC1978319

[ref44] Smith C. J. Renshaw P. Yurgelun-Todd D. Sheth C. (2022). Acute and chronic neuropsychiatric symptoms in novel coronavirus disease 2019 (COVID-19) patients: a qualitative review. Front. Public Health 10:772335. doi: 10.3389/fpubh.2022.772335 36033820 PMC9404694

[ref45] Velluti R. A. (1997). Interactions between sleep and sensory physiology. J. Sleep Res. 6, 61–77. doi: 10.1046/j.1365-2869.1997.00031.x 9377536

[ref46] Wang Y. Hunt K. Nazareth I. Freemantle N. Petersen I. (2013). Do men consult less than women? An analysis of routinely collected UK general practice data. BMJ Open 3:e003320. doi: 10.1136/bmjopen-2013-003320, PMID: 23959757 PMC3753483

[ref47] Wingrove J. Makaronidis J. Prados F. Kanber B. Yiannakas M. C. Magee C.. (2023). Aberrant olfactory network functional connectivity in people with olfactory dysfunction following COVID-19 infection: an exploratory, observational study. EClinicalMedicine 58:101883. doi: 10.1016/j.eclinm.2023.101883, PMID: 36883140 PMC9980836

[ref48] Wu T. Jia X. Shi H. Niu J. Yin X. Xie J.. (2021). Prevalence of mental health problems during the COVID-19 pandemic: a systematic review and meta-analysis. J. Affect. Disord. 281, 91–98. doi: 10.1016/j.jad.2020.11.117, PMID: 33310451 PMC7710473

[ref49] Yokoyama T. K. Mochimaru D. Murata K. Manabe H. Kobayakawa K. Kobayakawa R.. (2011). Elimination of adult-born neurons in the olfactory bulb is promoted during the postprandial period. Neuron 71, 883–897. doi: 10.1016/j.neuron.2011.05.046, PMID: 21903081

[ref50] Yus M. Matias-Guiu J. A. Gil-Martínez L. Gómez-Ruiz N. Polidura C. Jorquera M.. (2022). Persistent olfactory dysfunction after COVID-19 is associated with reduced perfusion in the frontal lobe. Acta Neurol. Scand. 146, 194–198. doi: 10.1111/ane.13627, PMID: 35467007 PMC9111206

